# Ultrastructural Characterization of the Lower Motor System in a Mouse Model of Krabbe Disease

**DOI:** 10.1038/s41598-016-0001-8

**Published:** 2016-12-05

**Authors:** Valentina Cappello, Laura Marchetti, Paola Parlanti, Silvia Landi, Ilaria Tonazzini, Marco Cecchini, Vincenzo Piazza, Mauro Gemmi

**Affiliations:** 1 Center for Nanotechnology Innovation@NEST, Istituto Italiano di Tecnologia Piazza San Silvestro, 12, 56127 Pisa, Italy; 2 NEST, Scuola Normale Superiore, Piazza San Silvestro 12, 56127 Pisa, Italy; 3 NEST, Istituto Nanoscienze-CNR, Piazza San Silvestro 12, 56127 Pisa, Italy; 4 Fondazione Umberto Veronesi, Piazza Velasca 5, 20122 Milano, Italy

## Abstract

Krabbe disease (KD) is a neurodegenerative disorder caused by the lack of β- galactosylceramidase enzymatic activity and by widespread accumulation of the cytotoxic galactosyl-sphingosine in neuronal, myelinating and endothelial cells. Despite the wide use of Twitcher mice as experimental model for KD, the ultrastructure of this model is partial and mainly addressing peripheral nerves. More details are requested to elucidate the basis of the motor defects, which are the first to appear during KD onset. Here we use transmission electron microscopy (TEM) to focus on the alterations produced by KD in the lower motor system at postnatal day 15 (P15), a nearly asymptomatic stage, and in the juvenile P30 mouse. We find mild effects on motorneuron soma, severe ones on sciatic nerves and very severe effects on nerve terminals and neuromuscular junctions at P30, with peripheral damage being already detectable at P15. Finally, we find that the gastrocnemius muscle undergoes atrophy and structural changes that are independent of denervation at P15. Our data further characterize the ultrastructural analysis of the KD mouse model, and support recent theories of a dying-back mechanism for neuronal degeneration, which is independent of demyelination.

## Introduction

Krabbe disease (KD)^1^, also known as globoid cell leukodistrophy, is a neurodegenerative disorder of the leukodistrophies family mainly affecting infancy-childhood and more rarely adulthood^2–4^. It is caused by the autosomal recessive mutation of lysosomal galactosylceramidase (GALC) enzyme, that leads to accumulation of galactosyl-sphingosine (psychosine), a potent lipid raft-associated neurotoxin^1, 5, 6^. Degeneration and death of myelinating cells are the main pathological hallmark of KD. More recently, psychosine accumulation was shown to influence neuron survival independently of the demyelination process^7^. Overall, both CNS and PNS are affected in KD patients^8^.

The only accepted treatment for KD in humans is hematopoietic stem cells transplantation, with scarce clinical outcome. Several other strategies have been proposed in a murine model for KD, the Twitcher mice (TWI)^9–11^, homozygous for the inactive form of GALC. Combination of cell therapy with gene therapy^12–14^, substrate reduction therapy^15^, pharmacological chaperone therapy^16^ and/or anti-oxidant therapy^17^, has resulted in a synergic therapeutic effect. Nonetheless, these strategies just afforded a slowdown of KD progression and a limited lifespan extension, suggesting that other targets should be identified and treated. Investigation of the cause-effect relationship between different overlapping mechanisms of the pathology is crucial to address this issue.

Mechanism of KD neural progression is currently debated. Recent studies proposed a dying-back mechanism for neuronal degeneration, independent of demyelination^7, 18^: indeed, Caspase3 (an apoptosis signal) is increased in KD sciatic nerves compared with spinal cords^8^. Furthermore, axonal loss in sciatic nerves occurs before apoptosis of cell bodies, even before clinical onset of KD symptoms^19^. These findings represent an alternative to older theories of the dying-forward and demyelination-dependent progression for KD^7^, based on the analysis of peripheral nerves ultrastructure^10, 20^. Additionally, the affection of endothelial cells-especially blood vessel walls architecture-in KD mice^21, 22^ further suggests that therapeutic approaches based only on the rescue of GALC activity in the brain may not be sufficient to cure KD disease.

In this study we exploited TEM to analyze the lower motor system of TWI mice in comparison with their wild-type (WT) littermates before onset and upon complete development of KD (P15 and P30, respectively). We have chosen the lower motor system owing to the presence of both CNS (spinal cord) and PNS (sciatic nerves), and also skeletal muscle. We started our analysis from sciatic nerves for consistency with previous findings^7, 20, 23^ and highlight here unprecedented details. Furthermore, we also analyzed the upstream spinal cord regions where somata of sciatic-nerves-forming motor neurons are located, and the downstream muscle innervated by sciatic nerve synapses. Our data unveil possible novel mechanisms taking part into KD progression that should be taken into account in the search for new therapeutic approaches.

## Results

### Ultrastructural analysis of the Sciatic Nerve in the late pathological stage (P30) of Krabbe disease

**Figure 1:**
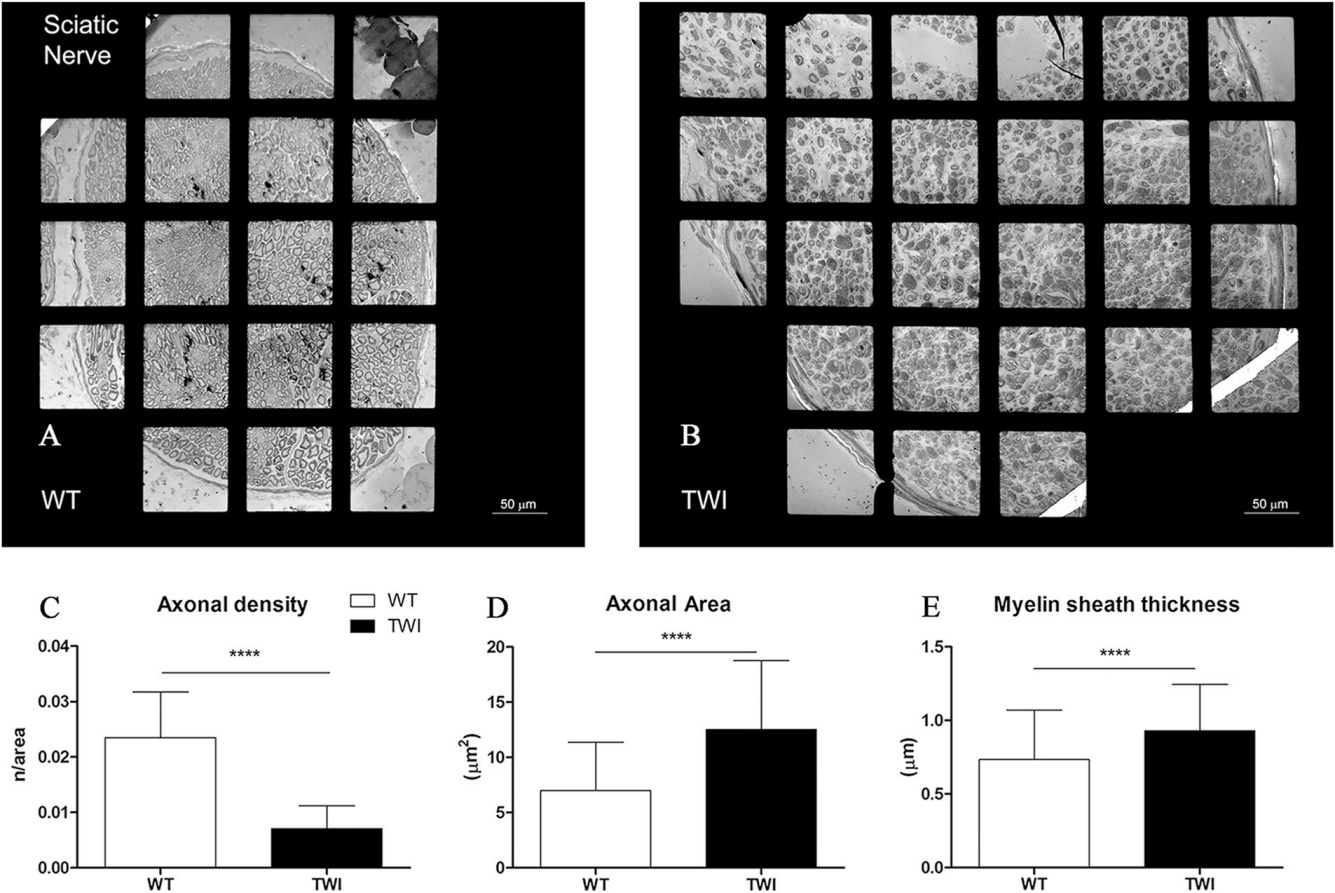
Qualitative and quantitative characterization of P30 sciatic nerves. (**A,B**) Representative micrograph of WT vs TWI sciatic nerve. The total size of sciatic nerve is increased in TWI samples. (**C**) Evaluation of axonal density (number of axons/axonal area). (**D,E**) Morphometric evaluation of axonal parameters.

We analyzed the ultrastructure of sciatic nerves just after trifurcation and built up 2D maps of the whole section of the biggest nerve branch (Fig. 1A,B). We observed a significantly larger nerve diameter in TWI mice compared with WT ones during the sampling. At the ultrastructural level, we observed that this effect depends on four main factors: (1) the presence of cells from immune system, especially globoid cells, an established hallmark of KD; (2) higher level of collagen fibrils (scar tissue); (3) enlarged cytosolic portion of Schwann cells (SCs); (4) increased empty space between axons. We then evaluated the density of myelinated axons within the main branch of the sciatic nerve, finding a significant reduction in TWI mice compared with WT littermates (Fig. 1C). All the morphometric data collected in each experiment are reported in Supplementary Figure 1A (WT: 0.023 ± 0.008 axons/μm^2^, n = 42; TWI: 0.007 ± 0.0004 axons/μm^2^, n = 47; ****P < 0.0001). We performed a morphometric analysis on myelinated axons (50 axons for each experimental group, Fig. 1D–E and Supplementary Figure 1B,C) based on the main ultrastructural parameters: area, myelin sheaths thickness (MST), diameter and perimeter. We found that the average size of axons is significantly larger in TWI sciatic nerves than in WT ones (Fig. 1D and Supplementary Figure 1B; WT: 7.00 ± 4.38 μm^2^, n = 50; TWI: 12.53 ± 6.24 μm^2^, n = 50; ****P < 0.0001). Additionally, perimeter and diameter evaluation indicates that TWI axons are swollen compared with WT ones (Supplementary Figure 1D,E). Furthermore, the MST of TWI sciatic nerves is larger than that of WT for every couple (Supplementary Figure 1C), in agreement with the average value (Fig. 1E; WT: 0.73 ± 0.34 μm, n = 50; TWI 0.93 ± 0.31 μm n = 50; ****P < 0.0001).

**Figure 2:**
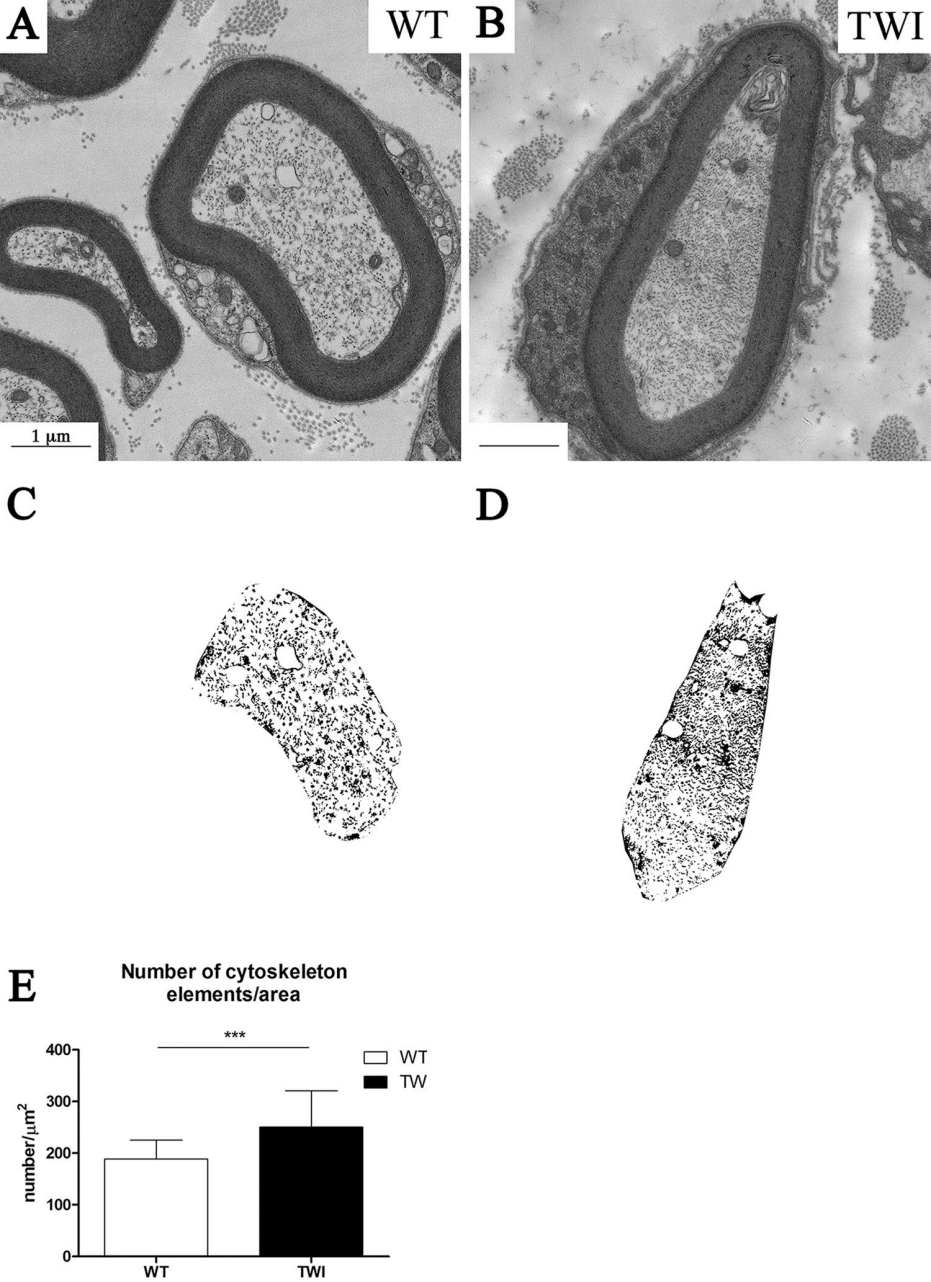
Axonal cytoskeleton density is increased in P30 TWI mice sciatic nerves. (**A,B**) bright field images of representative axons in WT (**A**) and TWI (**B**) sciatic nerves. (**C,D**) bitmaps obtained with the semi-automated method to compute axonal cytoskeleton density from the images in A and B, respectively. (**E**) Quantification of cytoskeleton elements density.

The organization and density of cytoskeletal components is altered in TWI sciatic nerves (Fig. 2A,B). Axonal microtubules and neurofilaments in TWI mice appear as a denser and more tightly packed structure compared with the corresponding WT. In order to quantify the axonal cytoskeleton density we developed a semi-automated method of image analysis. This method generates a bitmap of the neural axonal sections (Fig. 2C,D) and counts the number of particles (i.e. cytoskeleton cross-section dots) per axonal area (Fig. 2E). Our results indicate that axonal cytoskeleton in the sciatic nerve of TWI mice is more densely packed than the WT ones (WT: 189 ± 37 elements/μm^2^, n = 30; TWI: 251 ± 70 elements/μm^2^, n = 30; ***P = 0.0007). Due to this increased cytoskeletal density, the number of trapped mitochondria is larger in TWI mice than in WT littermates (Supplementary Figure 2B,C; WT: 0.4 ± 0.2 number/μm^2^, n = 30; TWI: 0.8 ± 0.6 number/μm^2^, n = 30; ***P = 0.0004).

**Figure 3:**
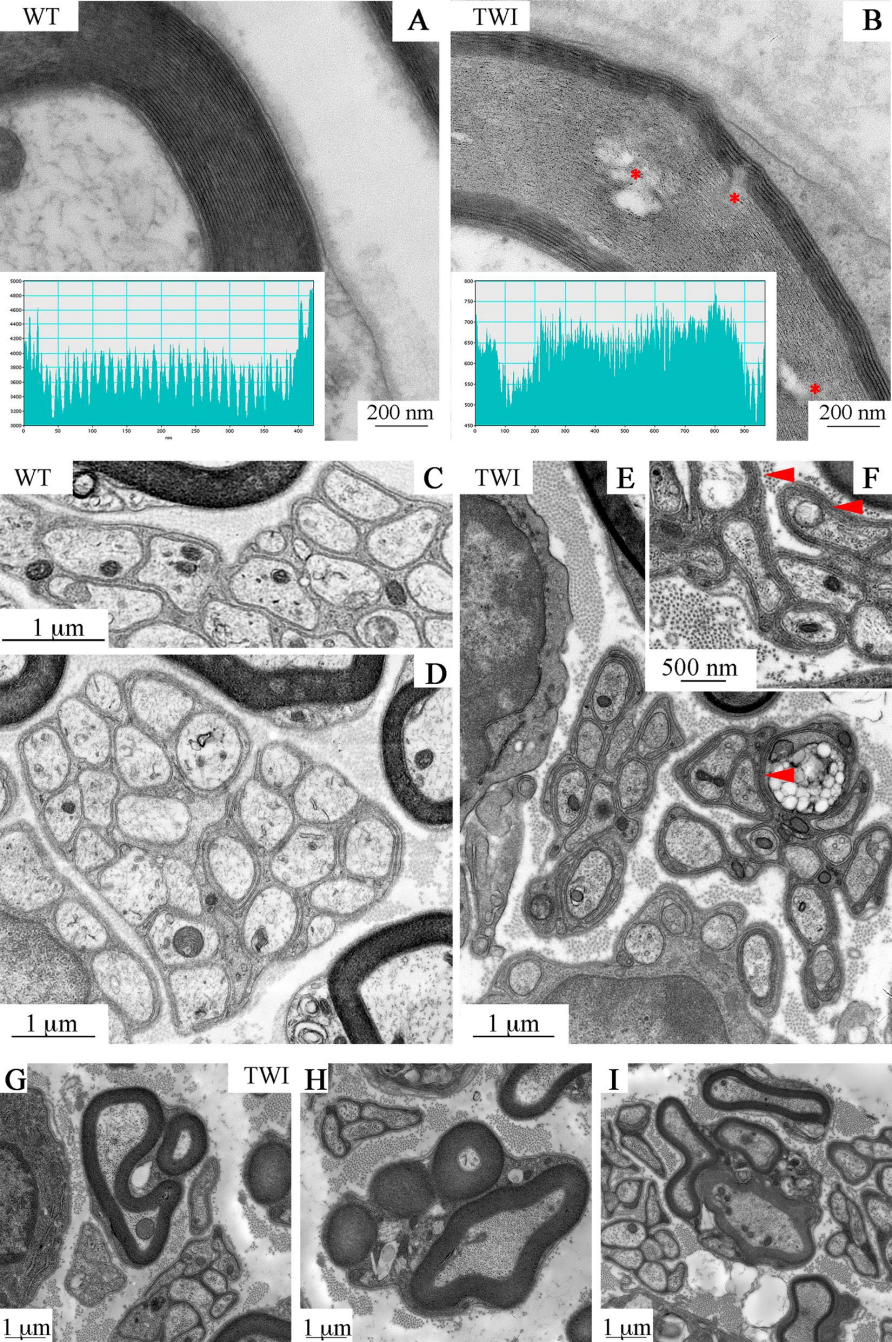
Myelinating and non-myelinating SCs in sciatic nerves of P30 WT and TWI mice. (**A,B**) Myelin sheath structure in WT and TWI samples; the insets show the image profile of a line crossing the myelin sheath. Gaps between consecutive layers are visible in TWI mice (B, *). (**C–F**) Remak bundles in WT sciatic nerves (**C,D**) and TWI ones (**E,F**). TWI mice Remak bundles show a denser cytoplasm compared to WT ones and a more complex winding (arrowheads). (**G–I**) Examples of multiple myelination processes in the sciatic nerve of P30 TWI mice. (**G**) type 1 MMP, (**H**) type 2 MMP, (**I**) type 3 MMP.

The cellular processes of SCs surround axons in a compact spiral of cytosol and membranes, forming myelin sheaths in the PNS. We observed a tight, well-organized myelin sheath of SCs around each axon in WT sciatic nerves (Fig. 3A). Conversely, sheaths in TWI samples are less organized and intermingled by empty spaces (* in Fig. 3B) and display orientation changes of adjacent myelin layers. TWI myelin thickness is significantly larger than WT one (Fig. 1E and Supplementary Figure 1C). However, number and thickness of a single myelin concentric layer (MCL) are conserved between TWI and WT samples (42 ± 2 rings; 11 ± 2 nm thickness). We then performed a more detailed investigation of the myelin-sheath organization to unveil the causes of this effect. We selected a subpopulation of WT and TWI neurons with area comprised between 3.5 to and 4.5 μm^2^, to avoid differences in myelin sheaths thickness related to axonal size. A gray-level profile of the image along a line crossing the myelin sheath shows disorganized structure of the MCLs through the entire TWI myelin sheath section (inset in Fig. 3A,B). In WT mice the profile exhibits a double-peak pattern in each wrap of the myelin sheath and the distance between peak-pairs, the height of each peak and the distance between the two peaks in each pair are constant throughout the sheath (Fig. 3A inset). Conversely, the profile across a TWI sheath is characterized by an irregular distribution of both peak intensity and spacing, which completely masks the double peak motif (Fig. 3B inset). We also observed that peripheral layers of the sheath–both in the inner and in the outer side–are generally more prone to alterations. This appears as a constant increase in the spacing between two consecutive myelin layers on the outer part of the sheath (Supplementary Figure 3A) or as a significant penetration into the axonal lumen by the few innermost myelin layers on the inner side (Supplementary Figure 3B).

Moreover, we observed an increased cytoplasmic area of Schwann cells in TWI axons. In WT axons, SCs are flattened around the myelin sheath and only small portions (mainly nucleus or perinuclear regions) are visible on the external side of the sheath (arrowheads in Supplementary Figure 4A). Instead, the TWI SCs appear enlarged (Supplementary Figure 4B) exhibiting degenerated organelles, mainly damaged mitochondria and swollen cisternae of endoplasmic reticulum (respectively dM and sR in Supplementary Figure 4C). Finally, some organelles -probably Golgi’s apparatus cisternae- are so degenerated that even at higher magnification it is possible to observe only a few discontinuous membranes (arrowheads).

Surprisingly, severe morphological alterations were also observed in non-myelinating SCs, which in sciatic nerves are those surrounding Remak fibers (Fig. 3C–F). Remak bundles in WT sciatic nerves are constituted of ten to twenty small-diameter axons (in the range of 0.5-1-5 μm) wrapped by an electrolucent envelope of non-myelinating SC cytoplasm (panels C and D). By contrast, we observed smaller groups in TWI mice, consisting of up to ten small axons surrounded by a more electron-dense envelope wrapping twice or three times each axon (arrowheads in panels E and F).

We also observed, in TWI sciatic nerves, a peculiar sub-population of myelinating cells close to Remak bundles that enclose more than one axon (panels 3 G-I). Since this multiple myelination cannot be due to preparation artifacts (see Methods) we can conclude that a variable architecture of myelination is a specific feature of the TWI sciatic nerve. We classified three different levels of multiple myelination processes (MMP, from the most to the least common): (1) one regular-sized axon close to one small axon (Fig. 3G); (2) one regular-sized axon and up to five small axons (Fig. 3H); (3) more than one regular-sized axons (Fig. 3I).

### Ultrastructural analysis of the spinal cord in the late pathological stage (P30) of Krabbe disease

**Figure 4:**
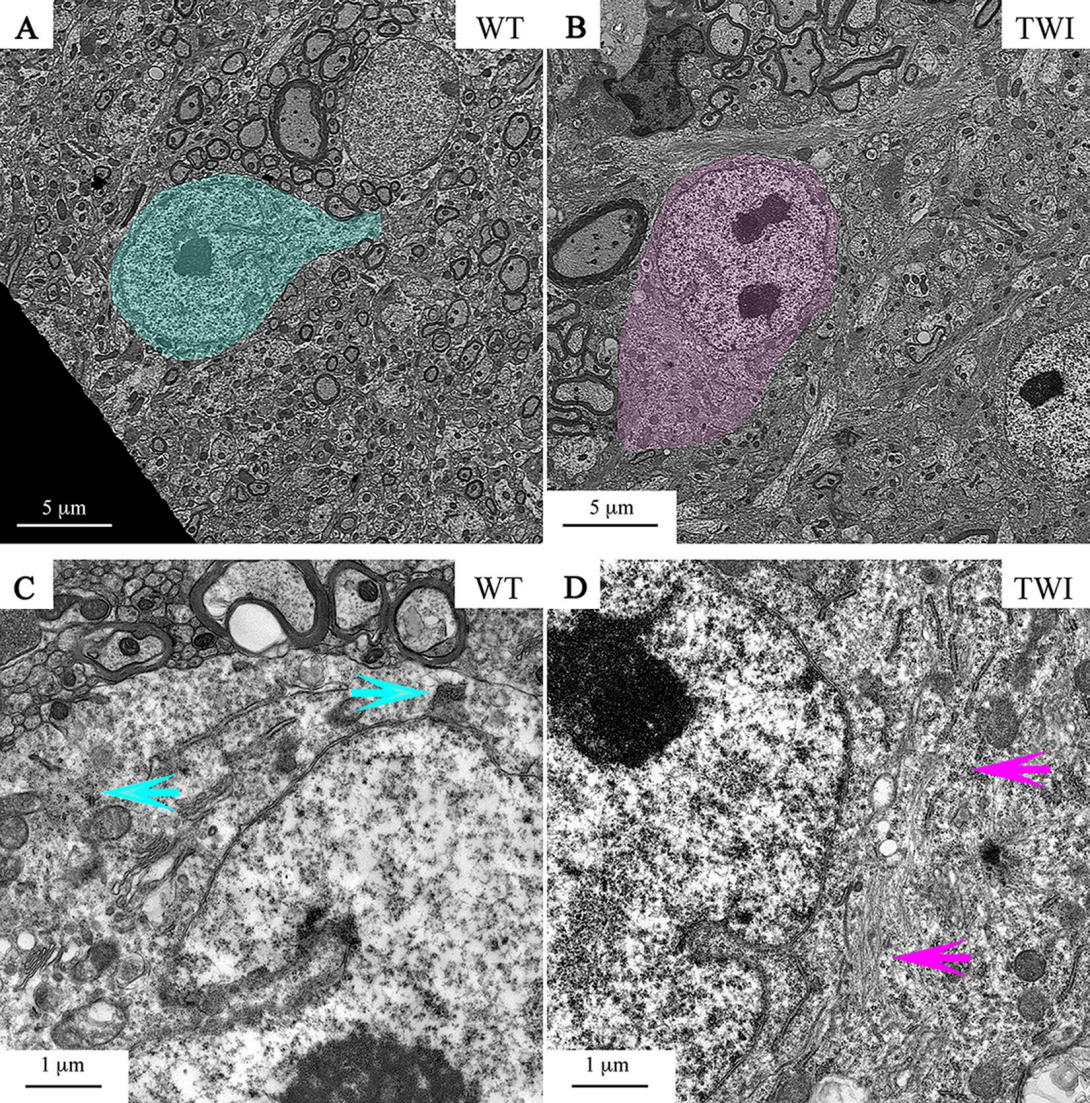
P30 motor neuron bodies in spinal cord. (**A,B**) MNs bodies (painted) in the spinal cord of WT and TWI. (**C,D**) Higher magnification of perinuclear regions of WT and TWI MNs. Arrows indicate normal (**C**) versus altered (**D**) actin cytoskeleton architecture.

We analyzed 10 to 20 motorneurons (MNs) bodies from TWI versus WT spinal cords. The ultrastructural features (size, shape, localization and number) of the MNs soma were similar in the two experimental groups (Fig. 4A,B). We only found a mild disorientation of actin filaments in the perinuclear region of TWI samples with respect to WT ones (Fig. 4D–light pink arrows) in about 5% of cells in only one mouse of the 5 TWI analyzed. However, WT MNs somata do not display such large aggregation of the cytoskeleton: we have always observed the usual phenotype of small bundles of actin filaments (Fig. 4C–light blue arrows).

**Figure 5:**
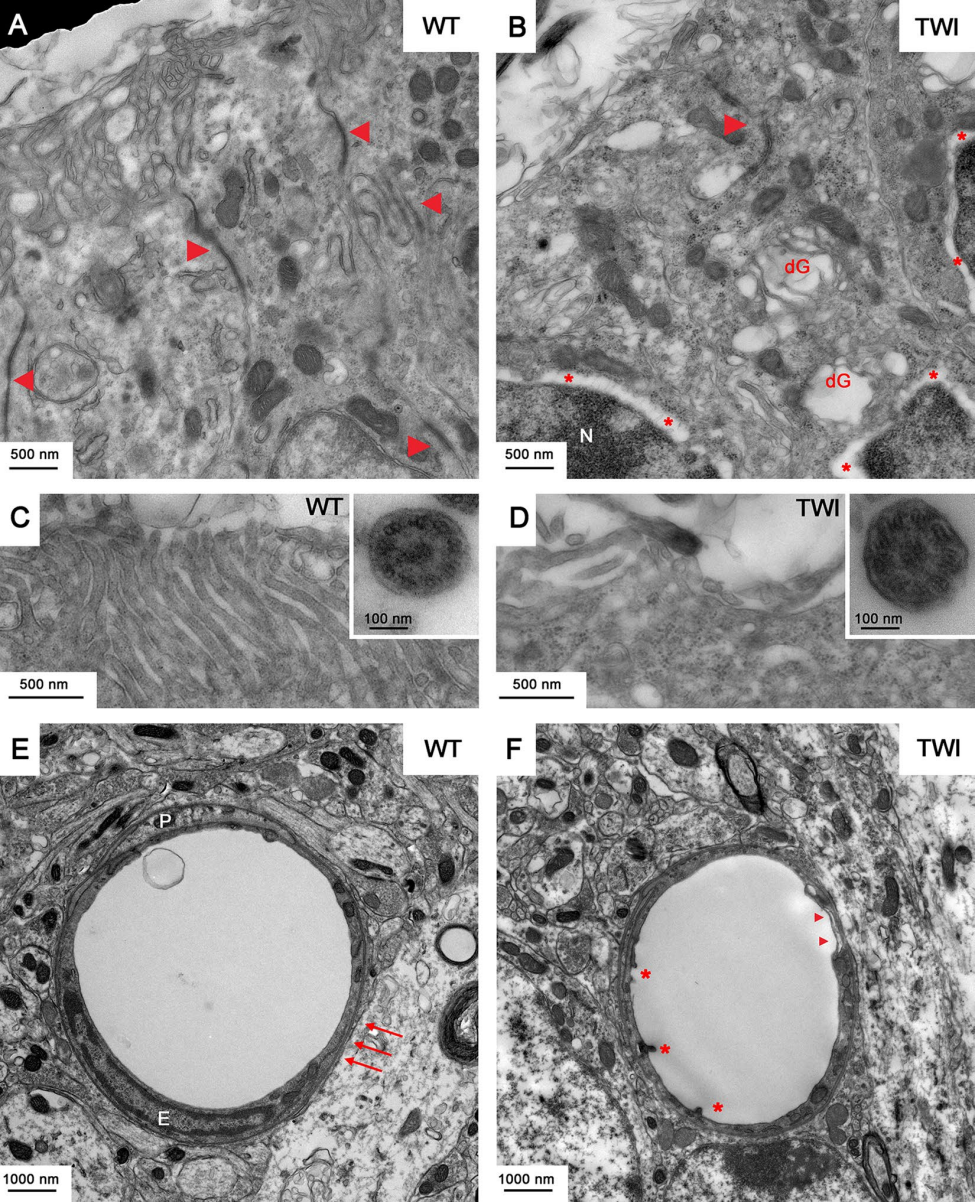
P30 ependymocytes and endothelial cells in spinal cord. (**A,B**) Representative TEM micrographs of the apical region of WT versus TWI ependymocytes. Arrowheads indicate cell to cell junctions; *indicate gaps separating the two lipid bilayers of the nuclear envelope; dG indicates degenerating Golgi’s cisternae. (**C,D**) Higher magnification images of microvilli and cilia (inset) in the apical region of ependymocytes of WT and TWI samples. (**E,F**) Architecture of WT versus TWI capillary; P: pericytes surrounding endothelial cells (**E**); arrows indicate evident basal membrane; *indicate intraluminal protrusions and arrowheads indicate thinner and discontinuous endothelial profiles.

Nucleus, nucleolus and nuclear envelope -and all other cytoplasmic organelles of TWI MNs are not different from those of WT MNs (Fig. 4C,D). In general, MNs soma of TWI mice are significantly less damaged than their respective axons (i.e. sciatic nerves). Infiltration of Globoid cells of the immune system, as well as the presence of lytic organelles in MNs bodies in the grey matter of spinal cords, arise only in extremely rare cases. Conversely, both epithelial cells of the ependyma channel and blood vessel wall endothelial cells are severely altered in their architecture in TWI mice spinal cord (Fig. 5). Conformation of the apical region of ependymocytes is normally well organized in WT animals, microvilli are uniformly distributed (Fig. 5C), and it is possible to observe the cytoskeletal structure of axoneme within motile cilia (panel C inset). The same cells of TWI mice (Fig. 5B) instead show an evident disorganization of cytoskeleton, degenerating organelles, mainly Golgi’s cisternae (dG), less-tight intercellular junctions, which are reduced in number and size (arrowheads in panels A and B). In addition, a separation between the two layers of the nuclear envelope can be observed (* in Fig. 5B), with the two bilayers (arrowheads in Supplementary Figure 5B) and the structure of the nuclear pores still visible (arrows in Supplementary Figure 5B). Microvilli are completely disorganized (Fig. 5D) while cilia and axoneme structures (inset in panel D) show no alterations. Concerning endothelial cells of the capillary wall, in WT samples we observed a continuous basal membrane (arrows in Fig. 5E), endothelial cells (E) with tight junctions and pericyte processes (P). In TWI samples instead we detected few regions with a thinner wall (arrowheads in Fig. 5 panel F) and several intraluminal protrusions of endothelial cells (*).

Both axonal and myelin sheath architectures are altered in the white matter of spinal cord of TWI mice compared with WT ones. We found that in several cases a clear separation is present between axons and their surrounding myelin sheaths. Cytoskeletal density follows the same trend observed in peripheral nerves indicating that neurofilaments and tubules are densely packed (Supplementary Figure 5C,D).

A morphometric analysis of the same parameters evaluated in sciatic nerves on those axons corresponding to the ventral root region indicates again that in TWI mice the value of axonal area and the MST are significantly larger than in WT ones (Supplementary Figure 5E–H).

### Ultrastructural analysis of the Gastrocnemius muscle in the late pathological stage (P30) of Krabbe disease

We focused our examination on motor units (MU: one motoneuron and all the fibers it innerves) of the Gastrocnemius muscle because this is a Fast twitch muscle predominantly formed by fast fatigable MU^24–26^. These have been well characterized to be more prone than other skeletal muscles bearing slower fibers (e.g. diaphragm), to degenerative processes in pathological conditions^27–29^.

**Figure 6:**
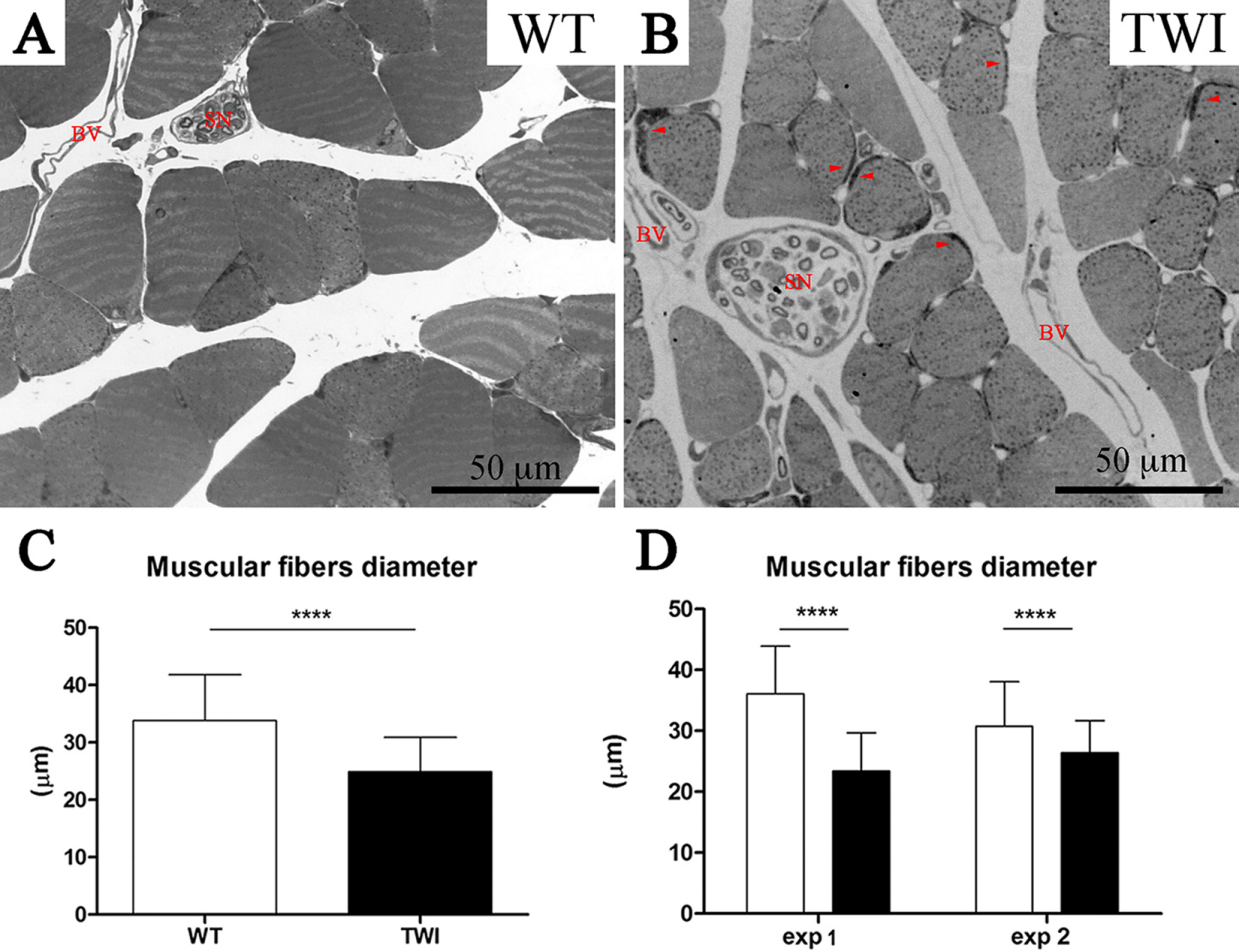
P30 gastrocnemius muscle fibers diameter. (**A,B**) Optical microscopy images showing the cross-sectional area of myofibers in gastrocnemius muscle of WT versus TWI mice. Arrowheads indicate higher mitochondria accumulation; SN indicate sciatic nerves and BV blood vessel. (**C**) Evaluation of mean values of myofibers diameter in WT and TWI mice. (**D**) Evaluation of fibers diameter in different mice show the same trend of reduction.

**Figure 7:**
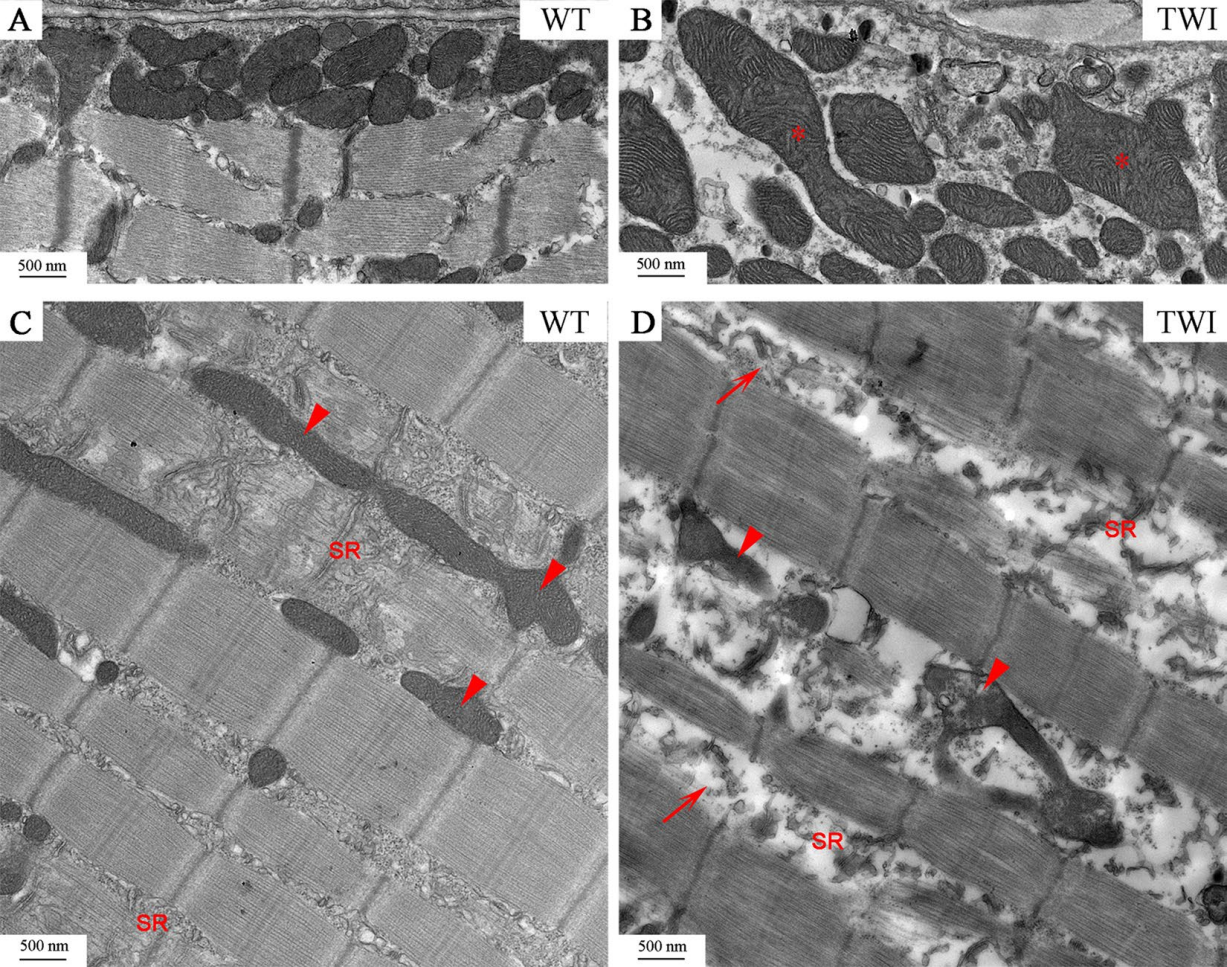
P30 Gastrocnemius muscle mitochondria and sarcoplasmic reticulum. (**A,B**) Representative micrographs of gastrocnemius subsarcolemmal mitochondria in WT and TWI mice. *Indicate swollen mitochondria. (**C,D**) Qualitative characterization of intermyofibrillar mitochondria. Arrowheads indicate representative mitochondria. SR indicates sarcoplasmic reticulum Arrows in D indicate T-tubules.

The first phenotype observed in the TWI mouse muscle is the reduction of muscular size, which is in agreement with the global weight loss observed^30, 31^. We quantified the area of single myofibers (Fig. 6C,D) and we found a significant reduction of TWI muscular fibers diameter (WT: 33.8 ± 8 μm n = 434; TWI 24.9 ± 6 μm n = 539; ****P < 0.0001). This was confirmed by cross section images of WT (Fig. 6A) versus TWI muscles (Fig. 6B). Interestingly, in those small fibers it is possible to identify darker regions (highlighted by arrowheads in panel B of Fig. 6) that likely correspond to the subsarcolemmal region of enlarged accumulated mitochondria (see Fig. 7 for more details).

We also observed that TWI subsarcolemmal mitochondria (Figs 6 (arrowheads) and 7B) are severely damaged and are twice or more as large as those observed in WT muscles (* in Fig. 7B). Intermyofibrillar mitochondria are altered in TWI muscles, although to a lower extent than subsarcolemmal ones, probably because they are less exposed to ROS and other oxidative species^32^; in this case, TWI muscle mitochondria share the same size of WT ones but differ completely in the ultrastructure of their inner membranes. Figure 7C shows typical images of WT interfibrillar mitochondria: arrowheads highlight the architecture of cristae and “SR” indicates the sarcoplasmic reticulum organized in cisternae and T tubules. This organization is completely lost in TWI interfibrillar mitochondria (Fig. 7D), in which both the outer and the inner membranes look discontinuous and mitochondrial cristae are undetectable. In addition the SR is evidently degenerated.

**Figure 8:**
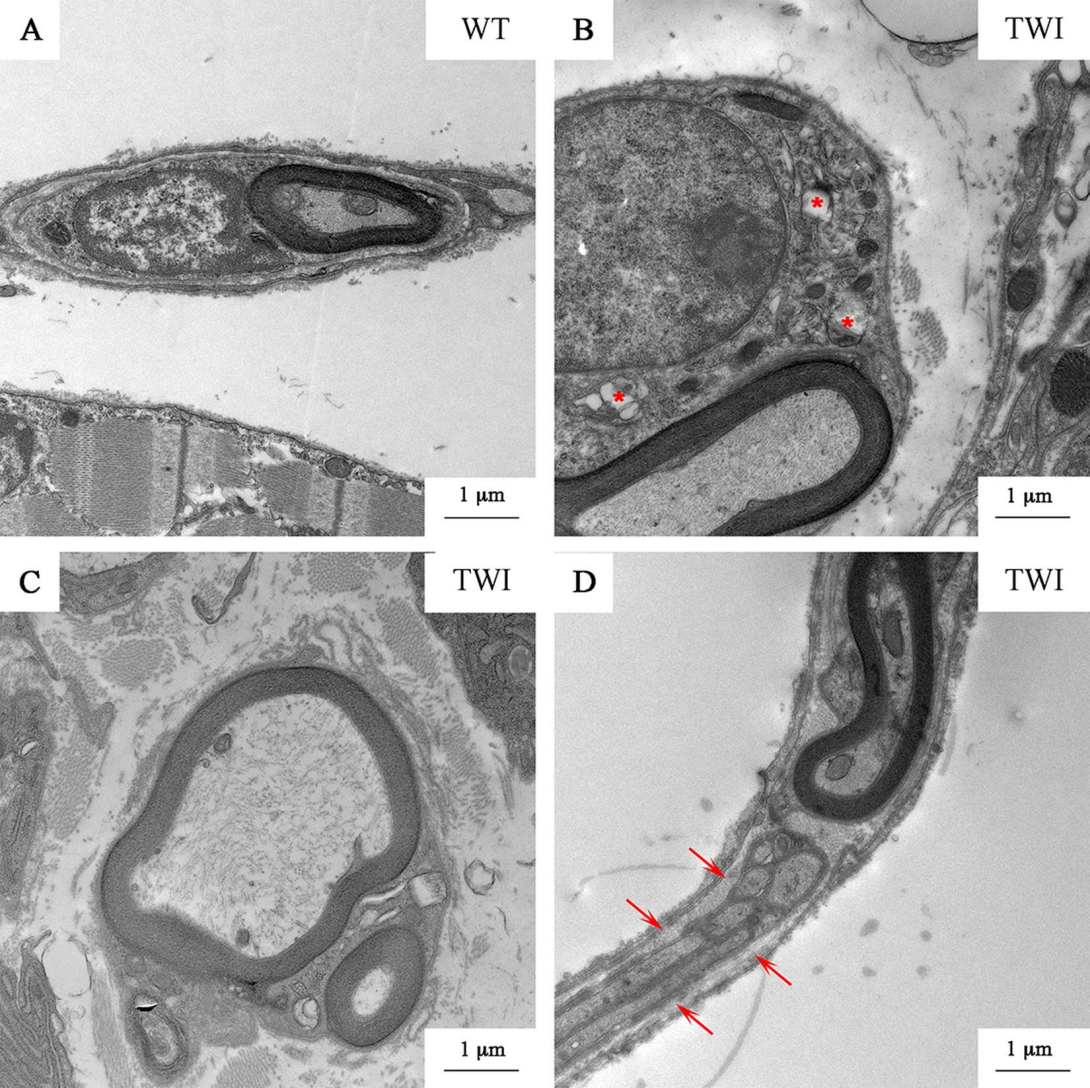
P30 nerve ending: axons and SCs. (**A**) WT nerve ending in gastrocnemius muscle. (**B**) SCs cytosolic compartment is enlarged and some damaged organelles (*) are visible; (**C**) MMPs; (**D**) bundles of non-myelinating SCs (arrows) surround myelinated axons.

For the description of SCs in skeletal muscle, we used the terminal branches of sciatic nerves (Fig. 8). These are constituted by at least one or two axons and the SCs covering the neuromuscular junction (NMJ). We observed no alteration of the myelin sheaths in the nerve terminals of TWI mice, but we found that the cytoplasmic portion of SCs surrounding the axons of the nerve terminals are larger than WT ones, and contain degenerating organelles like swollen mitochondria and swollen Golgi’s and endoplasmic reticulum cisternae. Thus, nerve terminals SCs share some degeneration features with sciatic nerve SCs (Supplementary Figure 4 *vs.* Fig. 8A,B). Characterization of SCs in this district highlights the presence of multiple myelination processes as observed at the proximal portion of the same nerve (Fig. 8C). Finally, analogously to what found in sciatic nerves (Fig. 3), we found structures similar to Remak bundles (arrows in Fig. 8D) into terminal branches. This does not occur in WT animals.

**Figure 9:**
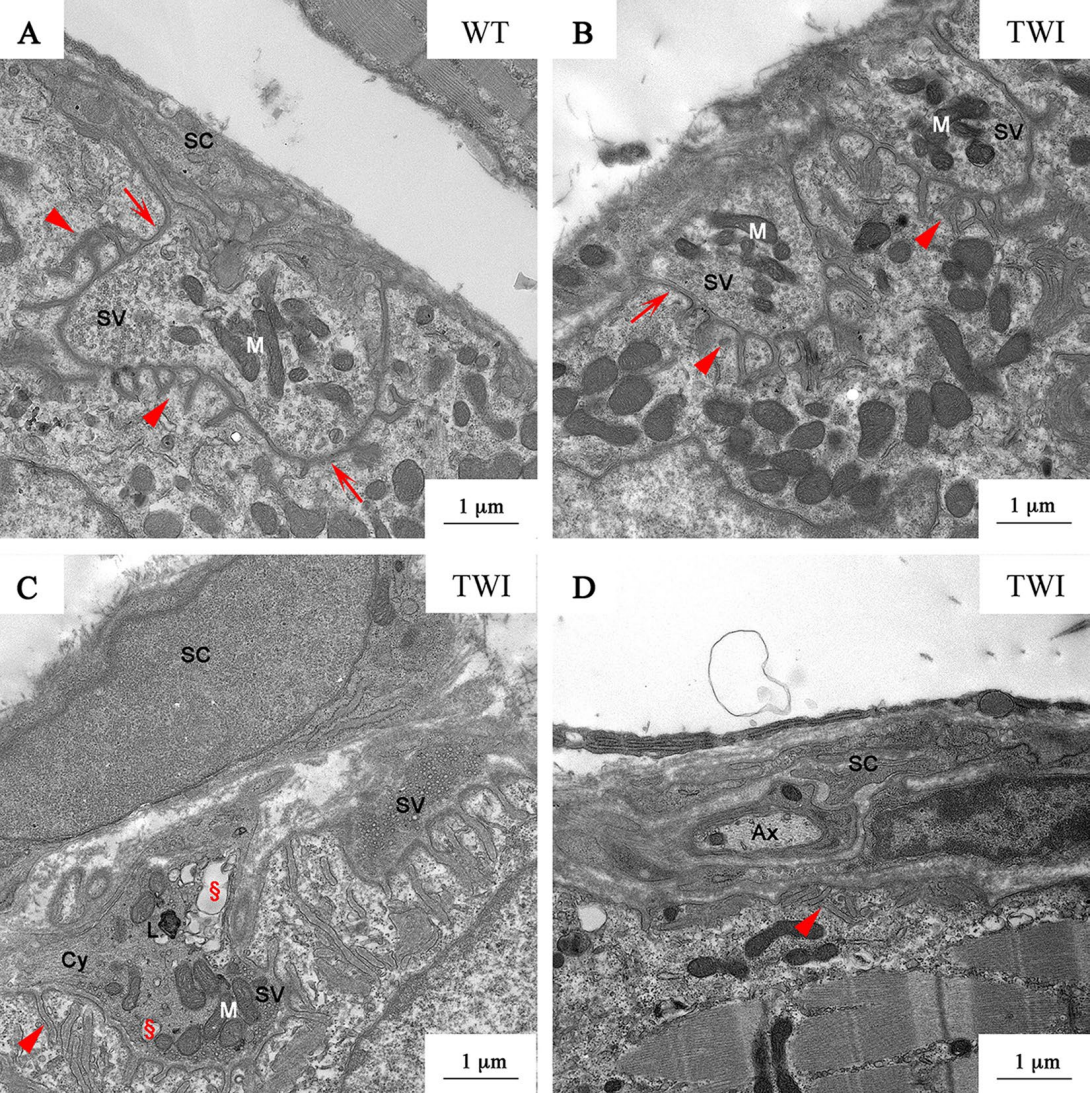
P30 neuromuscular junctions in gastrocnemius muscle. (**A**) NMJ in P30 WT gastrocnemius. SC: Schwann cell processes; SV: synaptic vesicles; M: mitochondria. Arrows and arrowheads indicate the primary and secondary folds respectively. (**B**) P30 TWI gastrocnemius NMJ type 1. (**C**) NMJ type 2; Cy: cytoskeleton elements; §: damaged organelles; L: lytic organelles. (**D**) Representative image of denervated NMJ (type 3) in TWI mice. Ax: axon.

To clarify the cause of the observed skeletal muscle alterations, we focused our attention on the ultrastructure of NMJs in the Gastrocnemius muscle. In WT samples (Fig. 9A), NMJs are characterized by the presence of several buttons of pre-synaptic terminals, with electrolucent cytoplasm and well-defined primary fold (arrows), where the presynaptic button is located. SCs and their processes surround the whole NMJ. Synaptic cleft thickness is regular, as well as the secondary folds (arrowheads) penetrating the post-synaptic element. In the buttons of NMJ we observed evenly distributed organelles (mainly mitochondria, M) and synaptic vesicles (SV) linked to actin filaments or docked to the pre-synaptic membrane. Conversely, we observed three different NMJ phenotypes in the TWI mice: (1) innervated NMJs similar to WT ones (Fig. 9A,B); (2) altered innervated NMJs (Fig. 9C); (3) denervated NMJ (Fig. 9D). The second group, which represents about two-thirds of the innervated NMJs, is characterized by the presence of smaller and flatter dark buttons (at least 3 for each NMJ) with a more electrondense cytoplasm. The number of SV is drastically reduced and those remaining are docked to the plasma membrane. The features of cytoskeleton (Cy), as observed in TWI sciatic nerves and spinal cords, indicate an increased density compared with WT or with unaffected TWI NMJ. In several cases we found the presence of lytic organelles (L) and/or degenerating organelles (§). Finally, the third group (Fig. 9D) is represented by denervated NMJs in which the presynaptic element is missing, the primary fold is flattened but the secondary folds (arrowheads) are still present. The whole structure, similarly to denervated junctions, is surrounded by SCs processes, enclosing also other axons (Ax). In TWI gastrocnemius the percentage of NMJ innervation, evaluated on their morphology, is 40% less with respect to the WT muscle.

### Ultrastructural analysis of the lower motor system in the early symptomatic stage (P15) of Krabbe disease

 In order to link the observed degenerations to the disease temporal progression we analyzed the ultrastructure of the same tissues in P15 animals.

Similarly to what found at P30, P15 TWI samples displayed: (i) enlargement of sciatic nerve diameter, increased dispersion of myelinated axons, and change of morphometric parameters describing axonal features of the proximal (Supplementary Figure 6) and distal (Supplementary Figure 8E,F) sciatic nerve. However, differences between the two experimental groups are usually milder than at latter pathological stages (Supplementary Figure 6C–G: Axonal density: WT: 0.031 ± 0.006 axons/μm^2^, n = 7; TWI: 0.024 ± 0.006 axons/μm^2^, n = 7; P = n.s. Axonal area: WT: 3.48 ± 2.09 μm^2^, n = 50; TWI: 4.32 ± 2.14 μm^2^, n = 50; *P = 0.0244. MST: WT: 0.62 ± 0.16 μm, n = 50; TWI 0.67 ± 0.19 μm, n = 50; P = n.s.; cytoskeletal elements density WT: 188 ± 58 elements/μm^2^, n = 10; TWI: 211 ± 58 elements/μm^2^, n = 10; P = n.s.; axonal mitochondrial density: WT: 0.6 ± 0.5 number/μm^2^, n = 10; TWI: 0.8 ± 0.4 number/μm^2^, n = 10; P = n.s.); (ii) Remak bundles structures smaller than WT ones, both in terms of size and number of sensorial axons per bundle (RB in Supplementary Figure 6A,B). Moreover, RB envelopes are more electron-dense and wrap each axon two or three times (Supplementary Figure 6H,I); (iii) reduced diameter of myofibers (Supplementary Figure 8; WT: 21.7 ± 5 μm n = 200; TWI 18 ± 4 μm, n = 200; ****P < 0.0001) and higher percentage of altered/innervated NMJ (Supplementary Figure 8I–J) in the gastrocnemius muscle.

Differently form P30 animals, we found that P15 TWI samples display: (i) no significant alteration in the MCL structure, as we did not find unmyelinated axons (Supplementary Figure 6A,B). Furthermore, although some swelling of SC cytosolic compartment was observed, this is likely a very early phenomenon as it is not associated with subcellular compartment degeneration; (ii) lack of cytoskeletal alteration in the perinuclear region of the MN body (Supplementary Figure 7A,B); (iii) unaltered state of ependymocytes as well as endothelial cells of capillary walls (Supplementary Figure 7C,F).

Thus the principal ultrastructural differences between P15 and P30 are summarized in Table 1.

**Table 1:** Comparison between early and late pathological stages in the lower motor system of TWI mice compared with WT littermates (−absence of signs; +mild differences; ++medium differences; +++severe differences).

	P15	P30
SPINAL CORD		
MN bodies		
number	−	−
size	−	−
shape	−	−
abnormal distribution of cytoskeleton	−	+
lytic organelles	−	+
subcellular compartments	−	−
Ependyma channel		
cell-to-cell junctions	−	+++
Microvilli	−	+++
Cilia	−	−
nuclear envelope	−	+++
Blood vessel		
thickness’ discontinouty	−	++
intraluminal protrusion	−	+
SCIATIC NERVE		
Nerve morphology		
swelling of nerve diameter	++	+++
infiltration of globoid cells	++	+++
scar tissue	++	++
enlarged cytosol	+	+++
empty spaces	+	+++
Remak bundles	+++	+++
Myelinated axons morphometry		
diameter	+	+++
area	++	+++
increased cytoskeletal density	+	+++
myelin sheaths thickness	+	+++
Multiple myelination process		
type 1	+	+++
type 2	−	++
type 3	−	+
GASTROCNEMIUS		
Fibers		
fiber diameter	+++	+++
alterated mitochondria	+	+++
alterated sarcoplasmic reticulum	−	+++
NMJ		
type 1	50%	27%
type 2	40%	33%
type 3	10%	40%

## Discussion

The therapies proposed so far to cure KD are only symptomatic and supportive. Increasing evidence points to KD onset and progression as a multifactorial pathology, in which several different cell types undergo degeneration. Understanding the causal and temporal links between these pathological phenotypes is crucially important to achieve an adequate understanding of this pathology.

The work presented here provides an ultrastructural characterization of the whole lower motor system of the TWI mouse model of KD disease. Neurons, myelinating and non-myelinating Schwann cells, ependymocytes, endothelial cells and muscular fibers were analyzed at a late pathological stage of disease progression (P30) and compared with those at an early stage (P15). We chose this experimental plan because it allowed us to study the progression of KD on two different scales. First, we gained details of the spatial progression of neural degeneration. Additionally, comparison between early and late disease stages allowed us to monitor the temporal progression of the pathology in the central and peripheral districts of the lower motor system, where the clinical symptoms first appear.

Initially, we directly addressed the question of KD neurodegeneration, an issue still debated since the 70s, when the TWI model was developed^11^. Recent theories point to a dying-back mechanism for neurodegeneration which is independent of demyelination in KD^7, 18, 19^; however, this view is in marked contrast with the older dying-forward view of KD progression^10, 20^. To reconcile these two hypotheses we investigated all-at-once three different districts: (i) the lumbar region of the spinal cord, focusing on the ventral horns of the gray matter where motor neuron soma are located; (ii) the sciatic nerve, made by axons of the same motoneurons, and (iii) the gastrocnemius muscle, which is innervated by the distal portions of the same motoneuronal axons. This allowed us to map the whole route of motor neurons from the CNS to the peripheral skeletal muscle, thus monitoring how the neural damage is related to the specific district investigated. Our results indicate that the bodies of TWI motor neurons are only slightly altered in the cytoskeletal organization (Fig. 4) in the late pathological stage, while no alteration is recognizable in P15 TWI samples. However, the observed change results in no other side effect in terms of size, shape and subcellular distribution of cytoplasmic organelles (Table 1).

Concerning the axonal compartment, we observed that while at P30 both MNs and SCs are severely affected (Figs 1 and 3), at P15 morphology of axons is already changed but myelination still unaffected. This observation confirms neural axonopathy to be an early hallmark independent of-and possibly the cause of-axonal demyelination, as previously described^19^. In addition, our data indicate that both cytoskeletal neurofilaments and microtubules are more densely packed in TWI than in WT axons at either early or late stages (Fig. 2 and Table 1). This is in agreement with previous works^7, 18^, where a reduction in phosphorylation levels of sciatic nerves neurofilaments was identified as the cause of increased cytoskeletal density. In our samples this phenomenon is accompanied by a larger number of mitochondria in the sections of TWI axons compared with WT ones (Supplementary Figure 2B,C). It is tempting to surmise that mitochondria are stuck as a consequence of axonal transport defects due to the dense network of both neurofilaments and microtubules (Supplementary Figures 2A and 6E). Indeed, impaired axonal transport has been related to several neuro-muscular disorders^23, 25, 27, 33^. Increased cytoskeletal density is also represented in the most distal portion of sciatic nerves: terminal branches and NMJ (Figs 8 and 9C). In the latter case increased cytoskeletal density characterizes about 30–40% of the total NMJ analyzed (type 2 or altered innervated NMJ, Fig. 9, Supplementary Figure 8 and Table 1), and is likely to be a prerequisite for impaired synaptic transmission and NMJ denervation. Taken together, all these findings indicate an unambiguous increase of neuronal degeneration as approaching the muscle, as most strikingly indicated by the cytoskeleton state. Thus, our results strongly support the recent theories of a dying-back and myelination-independent mechanism of KD progression^7, 19^, indicating that early reported TEM data lead to an incorrect description of KD progression. Presumably, these studies considered axons and myelin sheaths as a single element of degeneration^10, 11^. By contrast, more recent works demonstrate that psychosine acts separately and differently in neurons and SCs.

Despite the lack of damage in the MCLs at P15, we observed that TWI SCs display an enlarged cytosolic portion (Supplementary Figure 6B) compared with WT littermates and that this presumably anticipates the degeneration of cytoplasmic organelles at P30 (Fig. 3A,B and Supplementary Figures 4 and 6). Conversely, and unexpectedly, the alteration of non-myelinating SCs is already established in sciatic nerves at P15 (Fig. 3, Supplementary Figure 6H,I and Table 1), thus providing a key to explain the MMP. We underline that both in the sciatic nerve (Fig. 3G–I) and in the regions of nerve terminals inside the muscle tissue (Fig. 8C), MMPs were observed in close proximity to bundles of non-myelinating SCs that appear to be more electron-dense than typical Remak bundles (Fig. 3E–I and Supplementary Figure 6H,I). These are possible evidences, that at P15 the signaling of damaged axons (e.g. neuregulin1 type III/erbB^34–36^) drives the conversion of non-myelinating in myelinating SCs to afford an endogenous remyelination mechanism with a compensatory effect, as already described in tumorigenesis^34^. Furthermore, it was demonstrated that the distinction between myelinating and non-myelinating SCs is not absolute because a reversion between them could manifest in demyelinating pathology or upon proper signaling coming from neurons^37^. Further experimental work will be required to provide a conclusive answer to this hypothesis. However, a proper understanding of the molecular basis of MMP would be an invaluable contribution: indeed, this could eventually lead to the development of myelin restoring approaches in demyelinating diseases.

Our analysis also confirms that psychosine exerts its toxicity also in cells other than neurons and SCs^38^. Indeed, we found marked rearrangements in the spinal cord both in endothelial and epithelial cells at P30 (vessels and ependyma, Fig. 5 and Supplementary Figures 5A,B and 7C–F). Conversely, ependymocytes of TWI P15 mice do not show a different phenotype compared or with WT littermates (Supplementary Figure 7C,D) nor with WT cells at P30 (Fig. 5A–D). Interestingly, endothelial cells forming the capillary walls in P15 spinal cords (Supplementary Figure 7E,F) display discontinuity of the vessels and intraluminal protrusions at P15 of both experimental groups, and these are in turn similar to P30 TWI samples (Fig. 5F). This suggests that at late pathological stages, in TWI mice spinal cords, small vessels still exhibit an immature architecture compare with WT littermates. Such phenotype could be due to altered actin cytoskeleton and may be responsible for impaired vascularization and flow of cerebrospinal fluid in the spinal region, in agreement with previous observations^21^.

Finally, we provide here the first example of ultrastructural characterization of skeletal muscle in KD. Muscular damage in KD has been documented in TWI mice as an early symptom of disease progression with a general muscular weakness and tremors; however, analysis was limited to hind limb muscles and muscle innervated by cranial nerves^11^. Here we analyzed the TWI gastrocnemius muscle at P30 and found clear signs of cell atrophy compared with WT muscles (Fig. 6), in agreement with what recently reported^39^. However, we also identified a reduced percentage of innervation (from 90% at P15 to 60% at P30, see Table 1), as assessed by morphological parameters (Fig. 9 and Supplementary Figure 8). This apparent discrepancy could be due to the type of muscle analyzed: gastrocnemius (our work) and soleus-diaphragm^39^. Indeed, it should be considered that the gastrocnemius muscle has been reported as more prone to alterations than other muscles during muscular and neuro-muscular degenerative processes (e.g. ALS and dystrophy^26^).

Subsequent ultrastructural characterization of the gastrocnemius muscle shows that several structures of TWI muscles are severely damaged at P30, in particular some organelles as the sarcoplasmic reticulum and mitochondria (both subsarcolemmal and intermyofibrillar–Fig. 7). Accumulation of psychosine in lipid rafts and the high concentration of lipid microdomains in mitochondrial cristae and sarcoplasmic reticulum membrane are a possible cause of the observed phenotype. In fact, connection between mitochondria and endoplasmic reticulum is very strong and mediated by structures (mitochondria associated membranes) enriched in lipid rafts^40^. In this case, the skeletal muscle might represent a primary target of psychosine toxicity: psychosine could cause SR degeneration, and this in turn could lead to loss of calcium storage districts, finally resulting in reduced muscular strength. However, a high concentration of free oxygen radicals, possibly mediated by imbalance of calcium homeostasis and inflammation, should also be taken into account as a cause of the observed phenotype, as demonstrated in cellular and animal models of KD^6, 15^. Indeed, we believe that therapeutic actions aimed at reducing oxidative stress and increasing muscular strength should be encouraged in KD treatment, as this approach has already proved to be useful to rescue muscular damage in a model of ALS disease^27^.

Since at P15 structural alterations are still mild, it would be important to act therapeutically before this temporal window to stop further degenerative processes. Such an approach was already found to ameliorate the phenotype in a neurodevelopmental disorder like Rett Syndrome^41^. Importantly, at P15 we observed a significant reduction of myofibers diameter which cannot be only explained by a 10% of NMJ denervation (Table 1 and Supplementary Figure 8A–C): this observation, together with the P30 data described above, further suggests that psychosine exert a direct toxic effect on skeletal muscle.

In conclusion, the data reported here represent a novel, unprecedented ultrastructural point of view that will be possibly exploited further to finally achieve an aware view of what can actually be targeted in KD disease.

## Methods

### Animals

The Twitcher mouse colony (Twi^+/−^ C57BL6 mice; Jackson Labs) was generously donated by Dr. A. Biffi (San Raffaele Telethon Institute for Gene Therapy, Milan, Italy). Animals were maintained and used according to the protocols and ethical guidelines approved by the Ministry of Health, as per Italian law (Permit Number: 0004419).

Genomic DNA was extracted from the clipped tails of mice by Proteinase K lysis buffer as previously described^42^. The genetic status of each mouse was determined from the genome analysis of the twitcher mutation, as reported in ref. 31. TWI male mice at P30 and P15 and their WT male littermates were used for experiments, while the TWI-Het littermates for the TWI colony maintenance^31, 42^. Surgical procedures for fixation were performed under urethane anesthesia (Sigma, 0.8 ml/hg), and all efforts were made to minimize mice suffering.

### Electron microscopy

Postnatal day 30 (P30) TWI mice and their WT littermates (5 for each experimental group processed in 5 different experimental sessions, every TWI with its WT littermate) and one P15 TWI mouse versus its WT littermate were perfused with a fixative solution (4% paraformaldehyde and 0.1%–1%–2.5% glutaraldehyde in phosphate buffer, pH 7.4). Sciatic nerves, spinal cords and gastrocnemius muscles were dissected and post-fixed for 4 hours at room temperature in the same fixative solution.

Spinal cords were dissected in the lumbar region, isolating four 1-mm-thick sections in the lumbar enlargement region and the gastrocnemius muscles were cut in small portions, approximately 1 mm^3^ in volume. Sciatic nerves were processed without further sectioning.

The selected tissues were further treated for epoxy resin embedding as previously described^43^. Briefly, the samples were deeper fixed in 2–2.5% glutaraldehyde in cacodylate buffer (0.1 M, pH 7.4). After rinsing, specimens were post-fixed with osmium tetroxide (1%)-potassium ferricyanide (1%) in cacodylate buffer, rinsed again, en bloc stained with 3% uranyl acetate in ethanol, dehydrated and embedded in epoxy resin, that was baked for 48 h at 60 °C. Thin sections were obtained with an ultramicrotome (UC7, Leica Microsystems, Vienna, Austria) and collected on G300Cu grids (EMS). Finally, sections were examined with a Zeiss LIBRA 120 plus transmission electron microscope equipped with an in-column omega filter.

### Maps generation

Images of all the grid holes containing samples were collected at low magnifications (between 250X to 400X) and post-processed using Photoshop CS6 software (Adobe) to reconstruct the whole nerve section, as shown in Fig. 1. These low magnification micrographs were used for the evaluation of the distribution and the density of myelinated axons (manually-counted axons normalized for the area) within the specimens and for the evaluation of blood-vessels size and distribution.

### Ultrastructural characterization and morphometric analysis

Electron digital micrographs were used for the evaluation of nerve architecture and for the distribution of neurofilaments within axons.

The morphology of the myelin sheaths was fully evaluated for integrity, thickness, size and shape. Data were analyzed using the software Fiji.

### Semi-automated evaluation of cytoskeleton within axons

The evaluation of cytoskeleton density was performed on 10 images of correctly-myelinated axons for each experimental point among three different experiments. Furthermore, we chose those axons that showed at least one region of myelin concentric layers stacking in the myelin sheaths to check the orthogonal sectioning. After an automatic adjustment of brightness and contrast, axons were isolated from the surrounding myelin sheath and the axonal area evaluated. Cytoplasmic organelles were deleted from the micrograph and, following an automatic thresholding of the images the generation of bit-maps, the number of identified particle was counted and normalized for axonal area.

### Statistical analysis

We first evaluated the normality of each data distribution with Shapiro-Wilk normality test and then we used Mann-Whitney non-parametric test to check the difference between the experimental groups. Values are reported as mean, standard deviation, number of values of experimental group and value of statistical analysis.

In supplementary materials we reported the evaluation of single experiments repeated two or three times for a more relevant analysis.

## Electronic supplementary material

Supplementary Information
