# Supplementary material for: Ultrastructural Characterization of the Lower Motor System in a Mouse Model of Krabbe Disease
Source: Sci Rep. 2016 Dec 5;6:1. doi: 10.1038/s41598-016-0001-8 (PMC5431369; doi:10.1038/s41598-016-0001-8)
Supplement: Supplementary file 1 — Supplementary Information [file 41598_2016_1_MOESM1_ESM.pdf]

Full title: "ULTRASTRUCTURAL CHARACTERIZATION of the LOWER MOTOR SYSTEM in a MOUSE MODEL of KRABBE DISEASE".

Short title: KD ultrastructure

Valentina Cappello [1,*], Laura Marchetti[1], Paola Parlanti[1,2], Silvia Landi[3], Ilaria Tonazzini[3,4], Marco Cecchini[2,3], Vincenzo Piazza[1], Mauro Gemmi[1].

[1] Center for Nanotechnology Innovation@NEST, Istituto Italiano di Tecnologia Piazza San Silvestro, 12, 56127, Pisa, Italy

[2] NEST, Scuola Normale Superiore, Piazza San Silvestro 12, 56127, Pisa, Italy

[3] NEST, Istituto Nanoscienze-CNR, Piazza San Silvestro 12, 56127 Pisa, Italy

[4] Fondazione Umberto Veronesi, Piazza Velasca 5, 20122 Milano, Italy

[*] Corresponding Author

valentina.cappello@iit.it

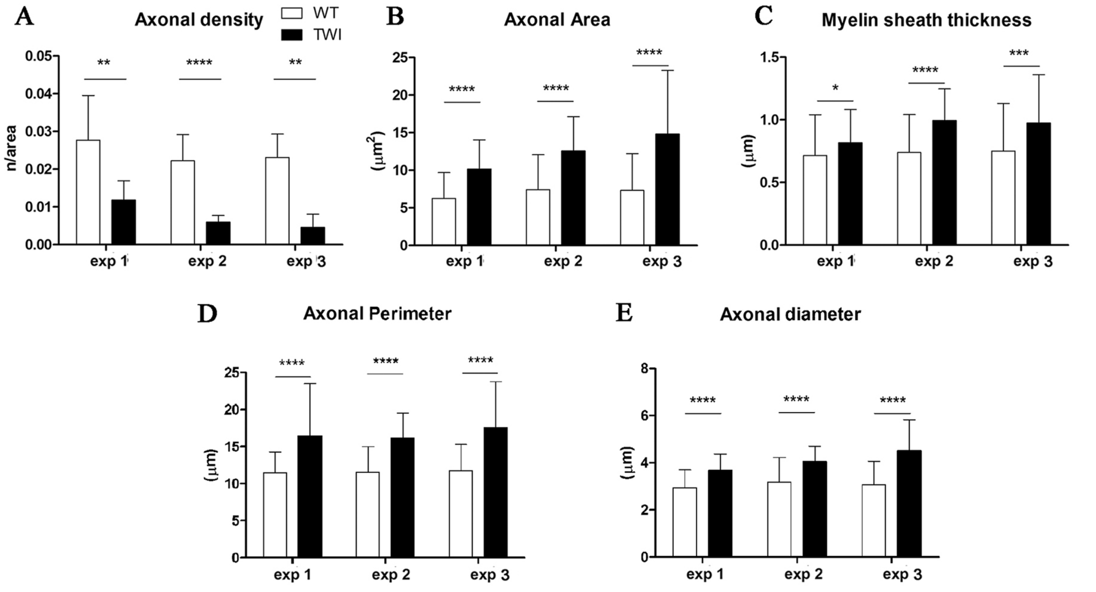

Supplementary Fig. 1. Quantitative characterization of sciatic nerves.

We evaluated morphometric parameters for each WT-TWI couple during the characterization of (A) axonal density; (B) Axonal area; (C) MST; (D) Axonal perimeter; (E) Axonal diameter. There is a similar trend of variation in each experiment.

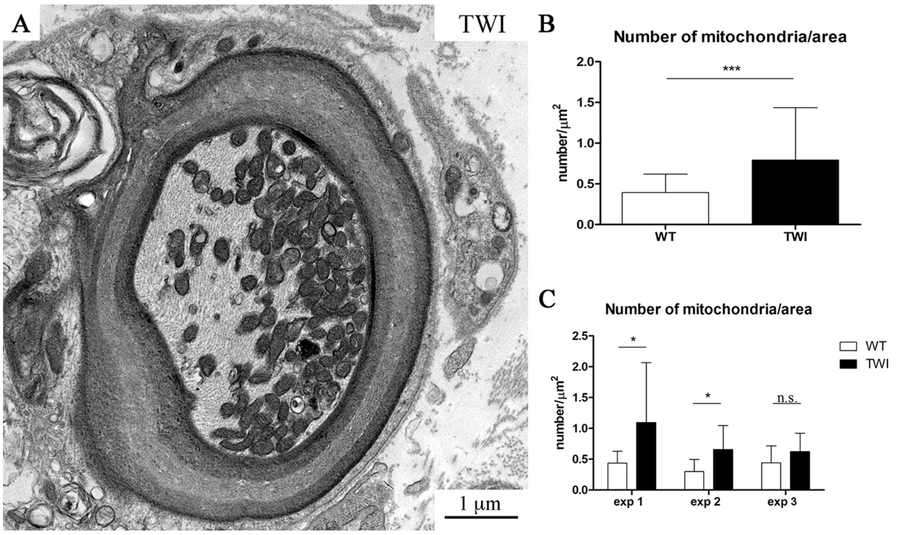

Supplementary Fig. 2. Mitochondrial traffic jam.

(A) Axonal cross section with an evident mitochondrial jam that we speculate could be linked to an axonal transport impairment due to the higher packaging of cytoskeletal elements in a TWI sciatic nerve axon. (B) Evaluation of the mitochondrial number normalized for axonal area; WT: 0.4 ± 0.2

mitochondria/μm^2, n =30; TWI 0.8 ± 0.6 mitochondria/μm^2, n =30; Mann-Whitney test P = 0.0004 ***. (C) Evaluation of the mitochondria number for each WT-TWI couple.

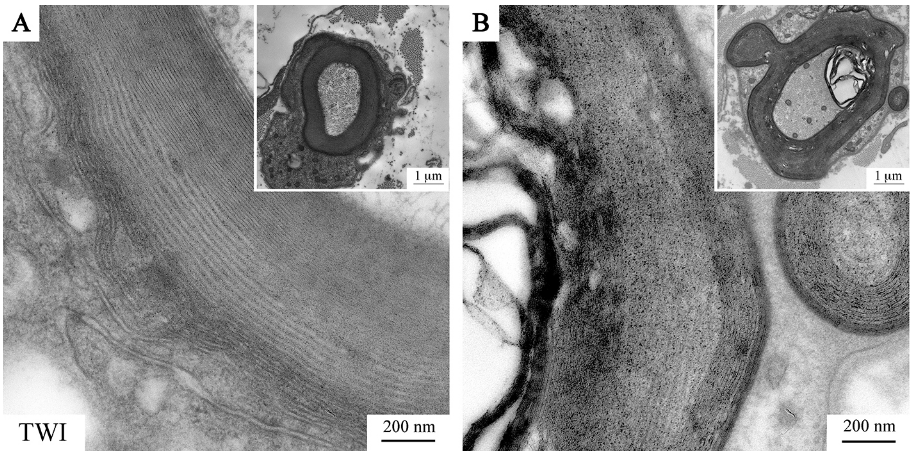

Supplementary Fig. 3. Analysis of P30 TWI myelin sheath disorganization.

Representative images of concentric layers of myelin sheaths in the sciatic nerve of TWI mice. Lower magnification images, in the insets, show the structure of a myelinated axon while the higher magnification clearly indicate that the deregulation of the staking could affect the axonal side of myelin sheath (B) as well as the outer portion of the nerve (A).

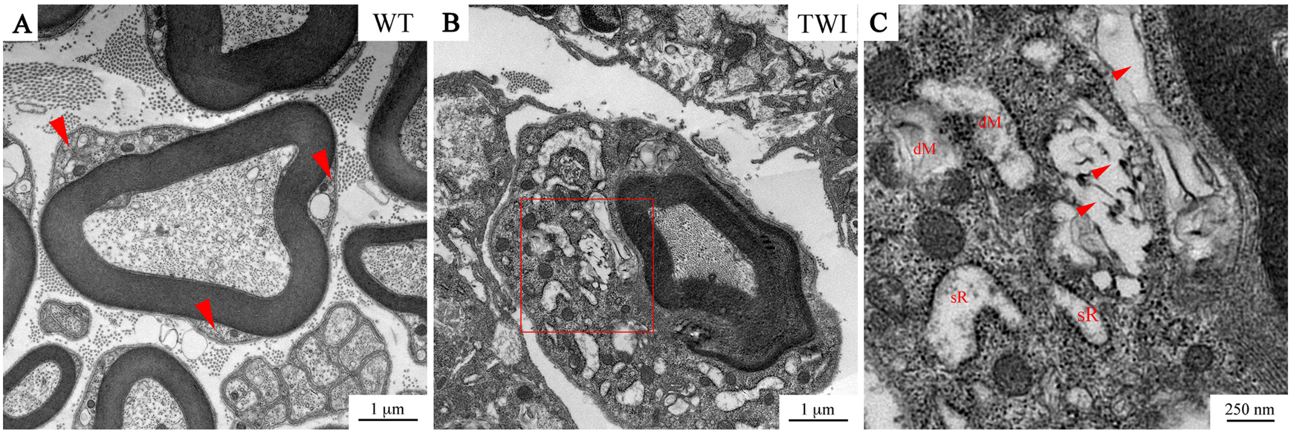

Supplementary Fig. 4 P30 myelinating SCs and their cytosolic organelles.

(A) Representative phenotype of a myelinating SC in P30 WT sciatic nerve. Just small areas (arrowheads) of the cytoplasmic compartment are enlarged and do not show any signs of alteration. (B) The cytosolic compartment of myelinating SC is enlarged in TWI mice sciatic nerve and clearly

affected. (C) Several organelles undergo degeneration like the swelling of endoplasmic reticulum cisternae (sR) or damaged mitochondria (dM), the latter resulting vacuolated and with disorganized cristae. In several cases we observed that the outer membranes of organelles are discontinuous as a possible effect of swelling (arrowheads).

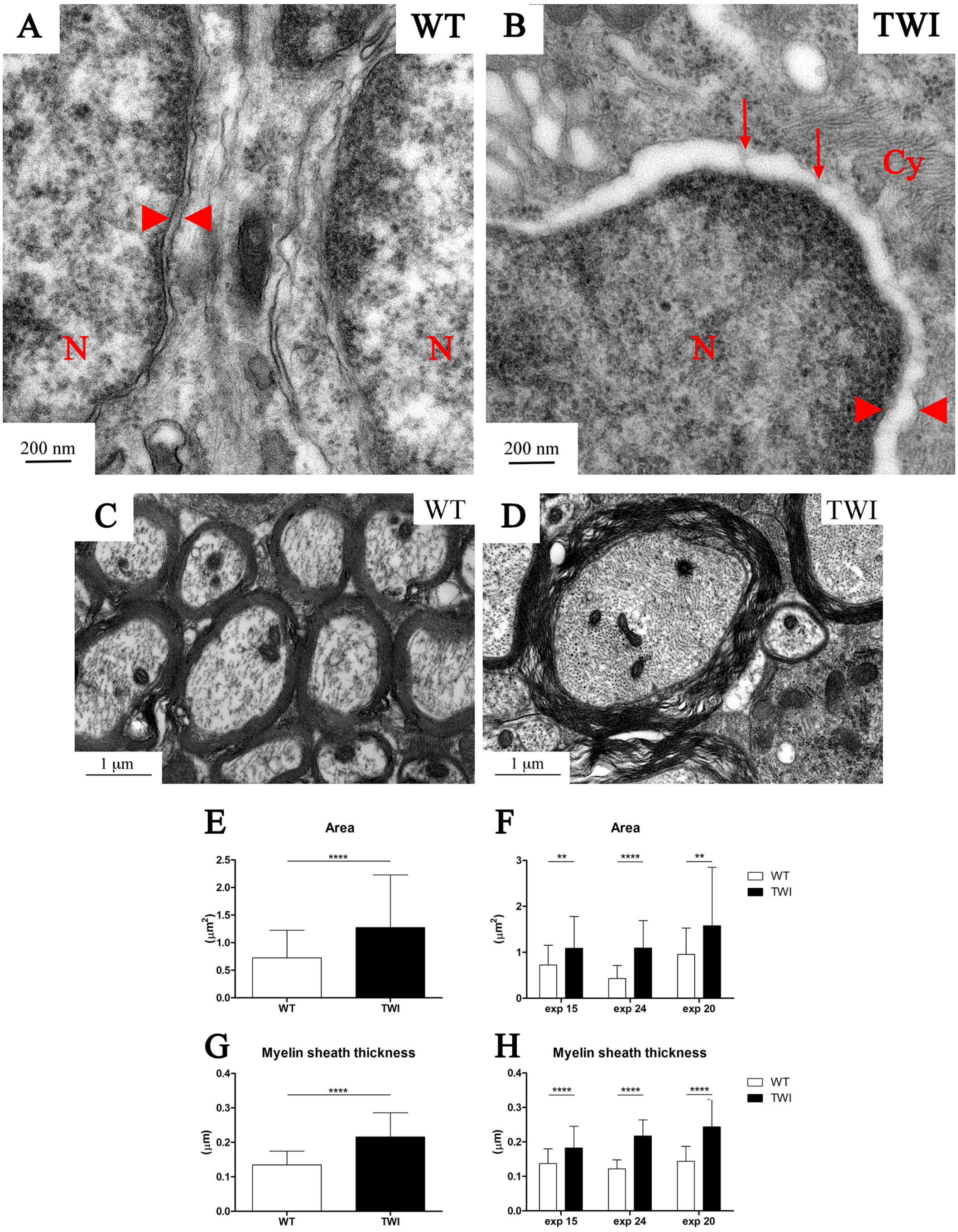

Supplementary Fig. 5. Alteration of nuclear envelope in ependymocytes and white matter axonal characterization. (A) The nuclear (N) envelope of ependymocytes in WT spinal cords, arrowheads indicate the two bilayers of nuclear envelope. (B) In the TWI spinal cord the two layers

of nuclear envelope are split and it is still possible to identify both the two bilayers of the envelope (arrowheads) and the membranous link of nuclear pores (arrows). Also the actinic cytoskeleton of the perinuclear region (Cy) shows an altered phenotype. (C) The ventral roots of spinal cords represent the region of motor nerves escaping and are characterized by the presence of small diameter axons with a well-organized myelin sheath, the presence of mitochondria and cytoskeletal elements. (D) Axons of the same region in the spinal cord of P30 TWI mice show several differences: an increased axonal size, thicker myelin sheaths, more densely packed cytoskeleton and an increased number of mitochondria in relation to axonal area. (E) The evaluation of axonal area shows a significant increase of this value in TWI mice at a late pathological stage (P30) compared to WT littermates: WT: $0.72 \pm 0.5\ \mu m^2$, n = 126; TWI: $1.3 \pm 0.9\ \mu m^2$, n = 126; Mann-Whitney test $P < 0.0001$ ****. (F) A similar effect has been observed in each experiment. (G) Myelin sheaths are thicker in TWI axon compared to WT ones: WT: $0.14 \pm 0.04\ \mu m$, n = 126; TWI $0.22 \pm 0.07\ \mu m$ n = 126; Mann-Whitney test $P < 0.0001$ ****. (H) Similar results for each couple of P30 TWI- control mice.

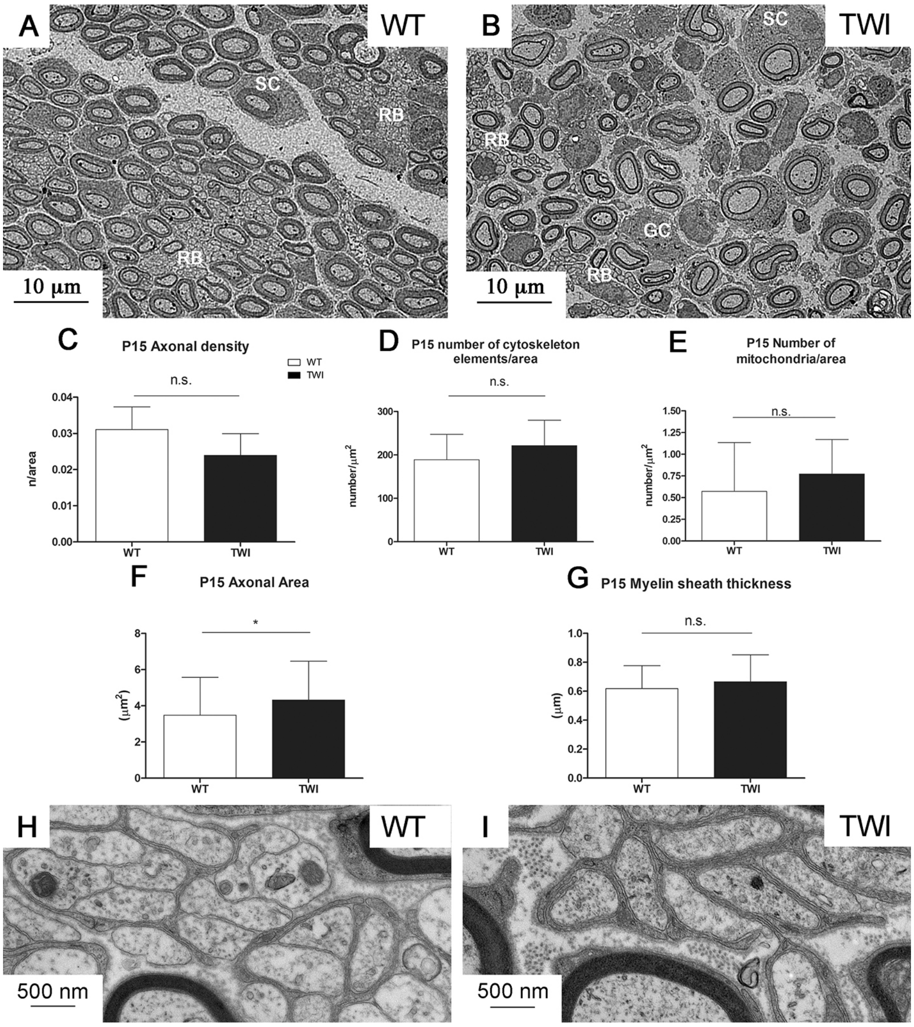

Supplementary Figure 6. P15 sciatic nerves

(A) Representative micrograph of P15 WT sciatic nerve of the main branch after nerve trifurcation. (B) The same region of TWI sciatic nerves of P15 animal. The density of axons is reduced in TWI samples because of enlarged SCs cytosolic compartment (SC) and because the presence of infiltrating cells (GC). The total area occupied by Remak bundles (RB) seems larger compared with P30 samples. (C) Evaluation of Axonal density (number of axons/axonal area). (D) Evaluation of cytoskeletal elements density. (E) Quantitation of axonal mitochondria. (F and G) Morphometric evaluation of axonal parameters. (H) Remak bundles in P15 WT sciatic nerves with their pale electrolucent matrix and with single winding around sensorial axons. (I) More complex and electrondense features are characteristic of P15 TWI Remak bundles.

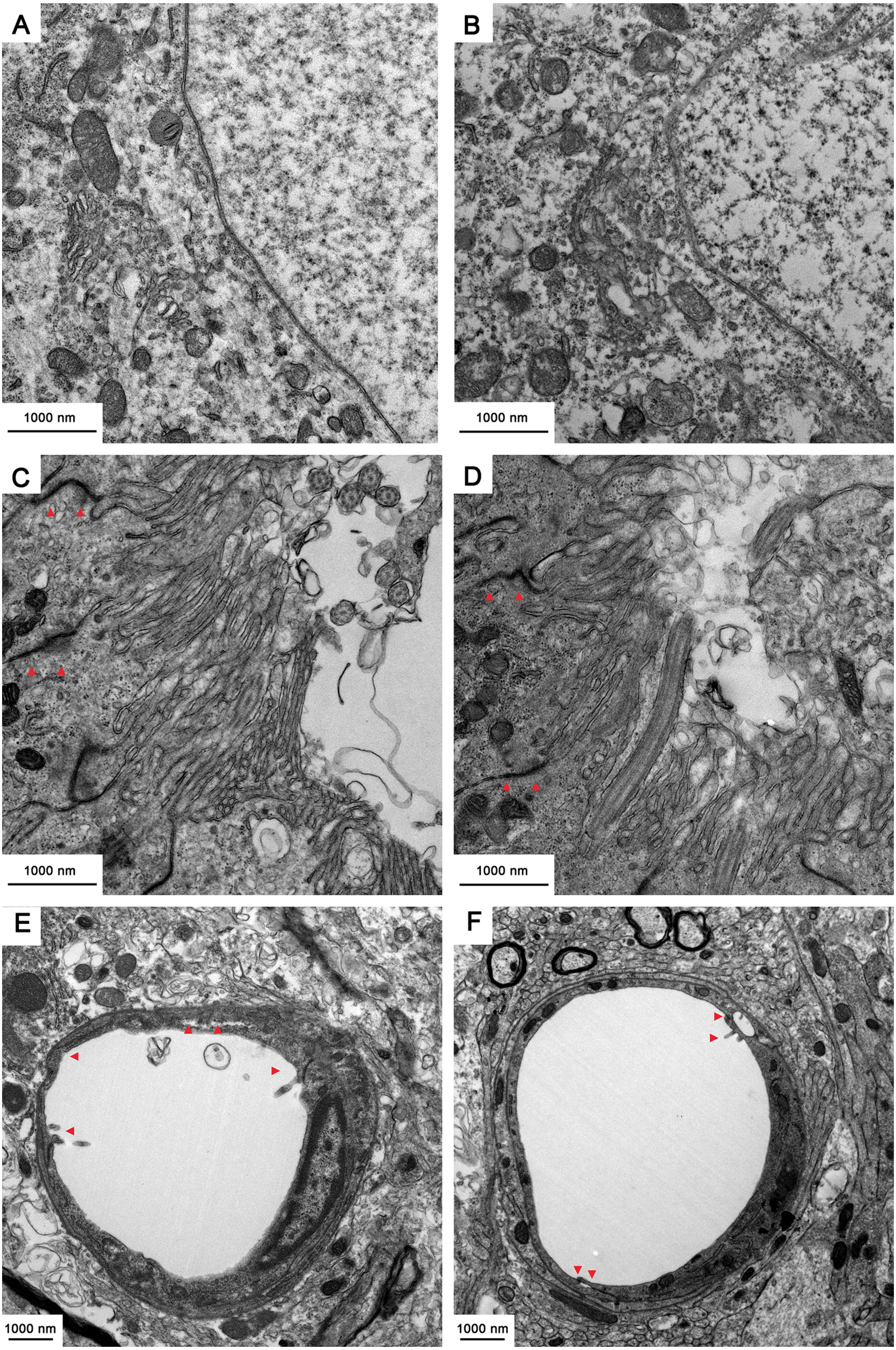
A
1000 nm
B
1000 nm
C
1000 nm
D
1000 nm
E
1000 nm
F
1000 nm

Supplementary Figure 7. P15 spinal cords

(A - B) Perinuclear region in MNs bodies of P15 WT (A) and TWI (B) spinal cords.

(C - D) Apical region of Ependymocytes in P15 WT (C) and TWI (D) samples with clearly visible cell-cell junctions (arrowheads), microvilli and cilia. (E - F) Endothelial cells forming capillaries wall in the grey matter of P15 spinal cord of WT (E) and TWI (F) mice. In both samples at P15 is possible to observe intraluminal protrusion and wall discontinuity (arrowheads).

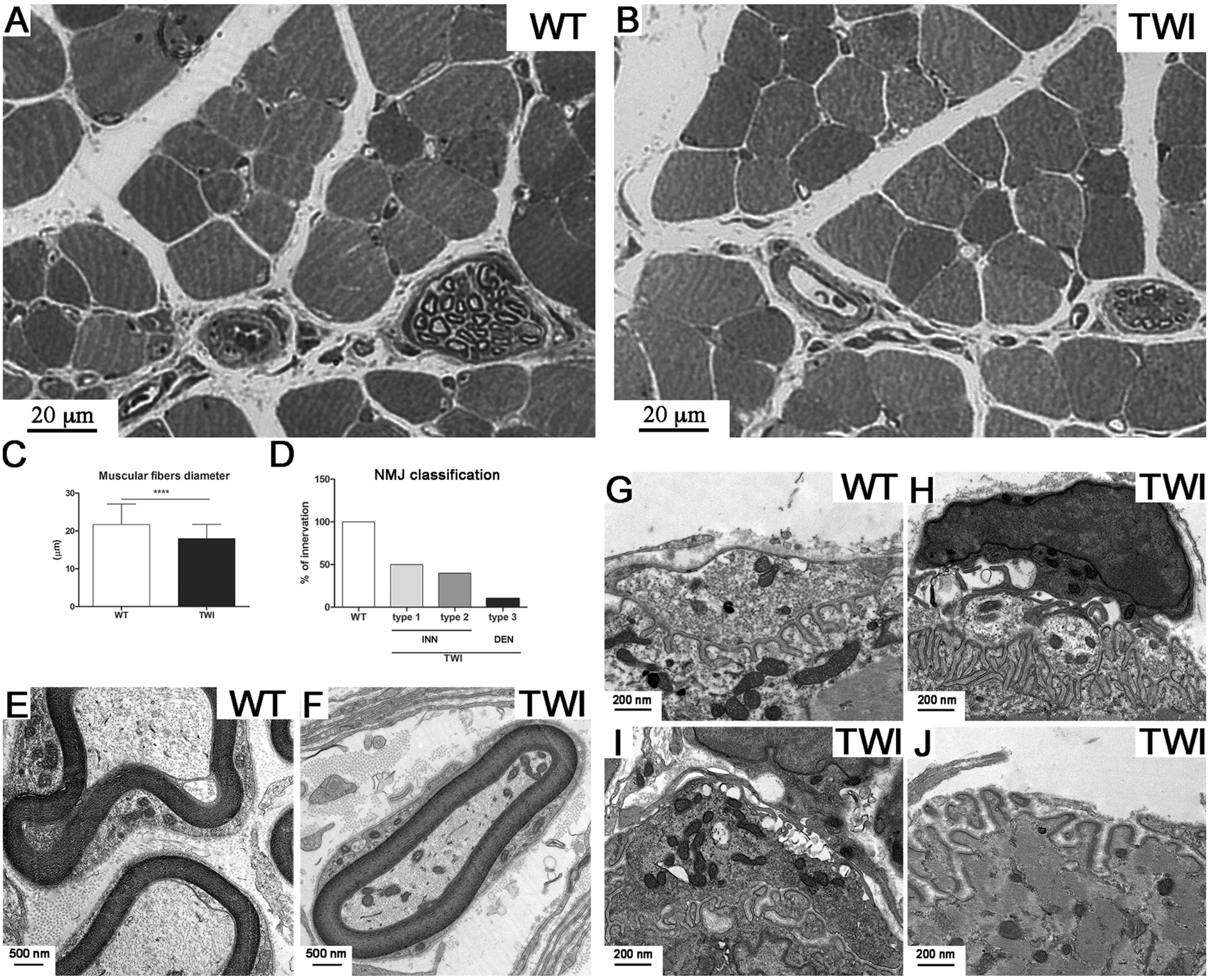

Supplementary Figure 8. P15 gastrocnemius muscle

(A - B) Optical microscopy images showing the features of myofibers in P15 gastrocnemius muscle of WT and TWI mice. (C) Evaluation of mean values of myofibers diameter in WT and TWI mice. (D) Evaluation of the percentage of innervation in P15 mice; even if the number of denervated junctions is 10%, the 40% of NMJ showed an altered phenotype. (E - F) Representative

micrographs of coronal sectioned nerve ending's axons in P15 animals; the density of cytoskeletal elements and the number of trapped mitochondria are increased in TWI samples (F) compared to WT littermates (E). (G-J) Morphological classification of the NMJs in P15 muscles. We identified an unaffected phenotype (H; Type1) similar to the WT architecture (G), a partially affected phenotype (I; Type2) of innervated junctions characterized by an increased density of cytoskeletal elements in the presynaptic buttons; and a denervated phenotype (J; Type3) in which the presynaptic element is completely lost.
